# Researching health inequalities with Community Researchers: practical, methodological and ethical challenges of an ‘inclusive’ research approach

**DOI:** 10.1186/s40900-015-0009-4

**Published:** 2015-08-13

**Authors:** Sarah Salway, Punita Chowbey, Elizabeth Such, Beverly Ferguson

**Affiliations:** 1 Health Equity and Inclusion Research Group, School of Health & Related Research, University of Sheffield, Sheffield, UK; 2 Centre for Health and Social Care Research, Sheffield Hallam University, Sheffield, UK; 3 Department of Nursing and Midwifery, Sheffield Hallam University, Sheffield, UK

**Keywords:** Community Researchers, Methodology, Participation, Health inequalities, Ethics, Identity

## Abstract

**Plain English summary:**

Public health research sometimes uses members of communities as researchers. These are called Community Researchers. The advantage of using Community Researchers is that it enables people who live in communities to participate in research by designing the research, gathering data and being involved in analysis. This ‘participatory’ approach also has the potential to reach communities that might otherwise not be included in research. There are few studies that report the experiences of Community Researchers who take part in such research. This study helps fill this gap by exploring the issues and challenges faced by Community Researchers involved in a study of health and poverty in ethnically mixed areas of east London, UK. Through the accounts of 12 researchers, the study reveals that being a community ‘insider’ had advantages: many felt they had been able to gain the trust of respondents and access people for the research that would have otherwise been missed. The role of Community Researcher was, however, difficult to manage with some researchers feeling burdened by their role and the increased knowledge they had about the lives of those in their community. In addition to the personal challenges for the Community Researchers, the findings raise various ethical and methodological issues that need consideration in participatory research.

**Abstract:**

**Background**

Inclusive research approaches are increasingly employed by public health researchers. Recent methodological development includes the engagement of Community Researchers (CRs), who use their knowledge and networks to facilitate research with the community with which they identify. Few studies have explored the experiences of CRs in the research process, an important element of any comprehensive assessment of the pros and cons of such research endeavours. We report here on the experiences of CRs engaged in a study of health inequalities and poverty in ethnically diverse and disadvantaged areas of London, UK.

**Methods**

We draw on the experiences of 12 CRs. Two sets of data were generated, analysed and integrated: debriefing/active reflection exercises throughout the 18-month research process and individual qualitative interviews with CRs, conducted at the end of the project (*n* = 9). Data were organised using NVivo10 and coded line-by-line using a framework developed iteratively. Synthesis and interpretation were achieved through a series of reflective team exercises involving input from 4 of the CRs. Final consolidation of key themes was conducted by SS and ES.

**Results**

Being an ‘insider’ to the communities brought distinct advantages to the research process but also generated complexities. CRs highlighted how ‘something would be lost’ without their involvement but still faced challenges in gathering and analysing data. Some CRs found it difficult to practice reflexivity, and problems of ethnic stereotyping were revealed. Conflict between roles as community members and investigators was at times problematic. The approach promoted some aspects of personal empowerment, but CRs were frustrated by the limited impact of the research at the local level.

**Conclusions**

Working with CRs offers distinct practical, ethical and methodological advantages to public health researchers, but these are limited by a range of challenges related to ‘closeness’, orthodox research structures and practices and the complexities of dynamic identities. For research of this type to meet its full potential and avoid harm, there is a need for careful support to CRs and long-term engagement between funders, research institutions and communities.

## Background

The past two decades have witnessed a considerable increase in public health and health services research that aims and claims to engage the subjects of research as active partners. Terms such as ‘Patient and Public Involvement’ (PPI), ‘user centred’, ‘participatory approaches’ and ‘community consultation’ are ubiquitous in academic research and health policy and practice circles in the UK and elsewhere (see for example, [1–5]). This move towards more inclusive research practices may offer particular leverage for those public health researchers concerned to understand and address the social determinants, and consequences, of ill-health.

Attempts to redress the power imbalance between researchers and the researched, to listen to the voices of marginalised people and to make research action-oriented are not new to researchers concerned with inequality [6]. Indeed, it is important to recognise that the UK has lagged somewhat behind other parts of the world in terms of effective citizen engagement in research, planning and development [7]. Nevertheless, the imperative to include and involve has never before been stronger, with government bodies, funding agencies, consumers of health research and organisations that claim to represent the interests of disadvantaged groups, all increasingly demanding and expecting that public health research be carried out in ways that are ‘inclusive’.

This methodological, and some would argue political, shift stems from at least two concerns. Firstly, it is increasingly recognised that many policy and practice interventions have disappointing or unpredicted outcomes due to a failure to adequately understand the broader context within which they are situated and particularly the ‘lived experiences’ of disadvantaged groups. The participation of groups who are the intended targets of policy is believed to enhance its acceptability, effectiveness and appropriateness [8]. Secondly, there is a growing acknowledgement that research itself frequently helps to shape and support socio-political structures that disadvantage and oppress [9].

Arguments in favour of inclusive research have commonly focused on increasing the ‘authenticity’ of accounts and thereby the usefulness of findings for policy or practice [10, 11] and/or the transformatory power of such research approaches for those disadvantaged individuals and groups that gain involvement [12]. However, while many have enthusiastically embraced the mantra of involvement and inclusion, it is increasingly recognised that neither the production of knowledge nor the empowerment of marginalised groups are unproblematic products of such research endeavours [13, 14]. In practice, a very wide variety of research approaches have been labelled as ‘user involvement’, ‘participatory’, ‘inclusive’ or ‘emancipatory’ [15, 16]. Importantly, the degree and extent of involvement varies widely, from consultation at certain times, to control throughout the project life cycle from topic identification through to knowledge translation [17]. Who has the opportunity and ability to become involved is also open to scrutiny, as Owen points out [16]. Engagement also presents possible risks for participants including stress, fatigue and stretched personal and financial resources [18, 19]. Indeed, approaches to public health research that aim to involve those outside academia as active partners raise a host of methodological, ethical and practical challenges that deserve close scrutiny. The present paper contributes to our understanding of the strengths and weaknesses of inclusive public health research by reporting on the involvement of Community Researchers (CRs) in a UK study.

The engagement of CRs represents one significant development in the inclusivity agenda. Such researchers have been engaged in a variety of public health research projects across a range of settings for several decades and are becoming more common in the UK in recent years [19–22]. A CR is an individual who is a member, and has direct experience of, a particular ‘community’^1^ and uses this knowledge to facilitate the gathering and interpretation of data. ‘Peer researchers’ and ‘lay researchers’ are other commonly used terms to describe research team members who are unlikely to be trained or experienced in research but are knowledgeable members of a distinctive community and who contribute to defining and conducting research projects. CRs are felt to bring a unique perspective and skills and contribute to the research process by doing the following: articulating the experience of the research participants in their own language, alerting outsider researchers to issues that may be overlooked, providing a link between the researchers and community members to increase trust, advising the researchers of appropriate ways of carrying out the research, and guiding the ways in which research products represent the community. Grander claims, relating to the empowering potential of such approaches, have also been made [11, 13]. Nevertheless, there has been relatively little published research to-date that has documented the processes and outcomes of CR involvement in public health research. Furthermore, as Warwick-Booth notes, academic papers that provide accounts of inclusive research rarely include the perspectives of local people engaged in such roles [21]. Boynton documented the experiences of CRs in a study of prostitution and noted both benefits (e.g. ownership of the research) and challenges including that the lack of immediate, tangible solutions to women’s concerns undermined participation [19]. Ryan et al. alert us to the danger of CRs not exploring particular issues during data collection that they or participants take for granted due to shared understandings [22]. Focussing on children and young people’s participation in research, Brownlie warns against its potential for ‘professionalisation’ and new forms of exclusion that may result [23]. There is clearly a need to know more about how CRs themselves enact and experience the role in different research contexts, in order to be able to improve practice, harness strengths and guard against harm.

By exploring the experiences of a group of CRs through their first-hand accounts, this paper provides an important addition to the literature on CRs and the practice of inclusive research more broadly. The specific objective was to describe and understand the experiences of a group of CRs with a view to identifying (i) practical lessons for effective working with CRs (such as issues to consider in designing training programmes, workload scheduling, payment levels and so on), (ii) the extent to which CR involvement increased the ‘quality’ of the main research study and factors that impacted upon this, and (iii) the extent to which CRs found the role to be a positive and empowering experience.

## Methods

The present paper draws on findings from an investigation into the behaviours and experiences of 12 CRs who were engaged in a study of long-term ill-health and poverty among deprived and ethnically diverse neighbourhoods in London, UK. This larger study, conducted 2005–2006, drew on a critical ethnographic framework [24] and aimed to describe relationships between long-term illness, poverty and social exclusion and to identify routes via which individuals and households could be better supported. Findings from the main study have been reported elsewhere [25].

CRs were engaged in the study primarily with a view to improving the rigour and relevance of the research. The assumption was that CR involvement would facilitate access to a wide range of research participants and aid interpretation of the data generated. The CRs were recruited through a variety of local networks with support from members of staff and local volunteers working with a community-based third sector organisation—Social Action for Health—with the requirement that they self-identified with one of four ethnic ‘communities’, White English, Bangladeshi, Pakistani or Ghanaian, and had strong local networks. Men and women were recruited with a range of ages, educational and occupational backgrounds. The CRs had no prior research experience but were provided with interactive training sessions and opportunities for collective reflection at intervals throughout the project: initial orientation (half day), training for phase 1 data collection (two days), training for phase 2 data collection (one day), analysis workshops (four days), and preparation for dissemination events (one day). The university researchers provided ongoing support throughout the project as well as encouraging peer support among the CRs by arranging paired working and group reflections. Owing to different levels of skill, experience, availability and interest, the nature and degree of involvement varied between individual CRs within the team. Nevertheless, all were involved in publicising the research project, identifying suitable locations and respondents and at least some direct collection, analysis and interpretation of data. Some CRs also engaged in the preparation of research products and the dissemination of findings.

Two sets of data were generated and analysed to address the present study’s objectives identified above. Firstly, throughout the project period, a series of mechanisms were used to capture information regarding the working processes of the research team. CRs themselves were regularly engaged in both one-to-one and group-based debriefing exercises focused on their own experiences and contribution to the research project. At the same time, the three university qualitative researchers included in their field diaries observations and reflections on working with the CRs. Secondly, all the CRs were invited to participate in an individual semi-structured interview with a researcher who was not connected with the research team, and nine such interviews were successfully completed and transcribed. The intention was to capture the perspectives of CRs themselves and the interviews explored: motivations for taking on the role of CR, perceived value of the work at the individual level, perceived contribution of the work to the wider community, relationships with the university researchers, relationships between CRs and other community members, challenges encountered during the work, and perceived longer-term effects of the work at individual and community level.

Data analysis and interpretation was undertaken in an iterative and participatory way. Prior to the detailed one-to-one interviews, four of the CRs were engaged in a one-day reflective analysis exercise with the university researchers in which the information gathered during the course of the project was discussed and consolidated. Through this exercise, key themes were identified and developed into a workshop presentation delivered at an external event. In addition, BF and ES, who had not been involved in the larger project, undertook an independent analysis of the interview material, performing both holistic memo writing and line-by-line thematic coding using the NVivo 10 software package. The interview findings were then integrated with the earlier material to produce the analysis presented here. In the end, resource and time constraints dictated that the final bringing together of the findings was undertaken by SS and ES, university researchers. CRs did, however, contribute to the development of the final analysis, commenting on earlier drafts of this paper. Ethical approval was obtained from the University of Sheffield, School of Nursing and Midwifery’s ethical review committee.

## Results

Our findings revealed that the involvement of CRs brought both advantages and complexities, with important implications for the CRs themselves, the university researchers and the products of the research. We organise our findings around key cross-cutting themes, each of which illustrates the ways in which practical, methodological and ethical issues were interrelated.

### Insider ‘closeness’

The CRs’ familiarity with their local communities offered significant advantages in terms of being able to recruit study participants who would otherwise have remained outside of the research. Many of the CRs drew on their diverse personal networks of family and friends to engage participants, enabling us to establish a respondent sample efficiently. The CRs themselves felt that their own identity—being physically and socially ‘close’ to the subjects of the research—gave them an advantage in recruitment. In one CR’s words: “If it’s about Ghanaians, then I’m a Ghanaian so I could relate to Ghanaians easily” (CR1).

Furthermore, most of the CRs argued that their involvement in the research brought invaluable lived experiences and ‘insider’ contextual knowledge to the project. They argued that their involvement both facilitated the posing of sensitive questions and allowed for a deeper interpretation of the data generated. In answer to the question ‘How important do you think it is that local people are involved as community researchers?’, CRs responded:

I think it is important. I’ve listened to the argument to say that non-local people can be just as effective and I think there is a point in that, but I think overall it is important because we’re dealing with inequalities here and the inequalities that we’re dealing with … non-local people may not even have realised that, you know, there are a lot of people in the local [area] who are here illegally and their lack of sensitivity is because they’re not [local] … [It] can often misread, you know, the situation [and] people’s reluctance … in bringing up [and] answering straightforward questions … it can be misinterpreted. (CR2)

I think it’s very, very important. You see they [CRs] have some knowledge that they can bring on board into the [research] and then they have contacts that they access … and they have some experiences that would be very relevant to the project, so I think they’re very important. (CR1)

Nobody knows a community like the people that live there… I definitely feel you’d miss a lot if you didn’t have local people involved. (CR8)

Though the CRs were able to encourage participation in some quarters, since ‘insider’ status was defined by a multiplicity of geographical, cultural and social identifiers, some CRs struggled to engage some sections of their ‘community’. In one case, social class and migration status were identified as key characteristics that demarcated social distance between the CR and the participants and undermined an open interview:

Ghanaians have a tendency not trusting each other especially with those who do not have right of abode in this country will be suspicious with any affluent Ghanaian, you know, coming … you know, telling them things and asking them questions. If you’re looking at it also from the point of view that … that from one social class to the other, as you were asking earlier, then they will only have to say why … why is somebody like me coming to, you know, interview them …Although I’m a Ghanaian, I’m a stranger in their home. (CR1)

In another example, a female CR reported not wishing to interview men from her ethnic community after a negative previous experience in which a male respondent dominated her and was generally difficult. In another case, a lack of strong personal networks resulted in the CR adopting an inappropriate strategy in an attempt to recruit participants.

I ended up going for a couple of half days just on the streets in Tottenham, stopping passers-by … looked like they might be ill or … and I found that hard work, going up to strangers and suddenly asking them personal questions, but I didn’t particularly enjoy that at all. (CR8)

More generally, CRs recognised that being ‘too close’ could in fact undermine some respondents’ willingness to speak freely, with suspicion sometimes being a barrier to rich interview data. Similarly, CRs recognised that though shared ethnic identity might provide a CR with useful insight and understanding, such identities are contingent, fluid and intersected by other factors so that ‘insider knowledge’ could not be taken for granted.

Interviewer: So is it particularly important for members of ethnic minority groups to be involved as [community researchers] …?

CR2: Yes. Yes, I do … And it’s not just every … any ethnic minority person. I think you want somebody with that experience, not somebody who qualifies by ethnicity, but somebody who qualifies both ethnicity and also experience and knowledge.

While the CRs were able to enter their communities and gain access to individuals more easily than the university researchers, being close to the community had some adverse consequences for the CRs. Listening to difficult stories, learning about previously unknown events in the community and being powerless to change things were identified by some CRs as difficult to cope with.

It was difficult when they were getting emotional and then I started feeling a bit tearful and tried to hold back my emotions and thinking, “Oh my god, I’m a community researcher. I shouldn’t be doing this.” (CR4)

Respondent concerns regarding confidentiality of information were noted by some CRs, and they felt that some individuals were reluctant to speak to them in detail about sensitive issues. Areas of sensitivity included issues around residency status, welfare benefits and some personal information.

Mostly they opened up, but on a couple of occasions, maybe, they didn’t open up because they thought, “Where is she going to give this information to? What is she going to do?” in terms of status and asylum and all that kind of stuff. (CR4)

In the community lots of … lots of people they just … they don’t want to lose their benefit or their anything, so they just … they think like yeah, “Let us be like this. Don’t need to talk about this.”(CR5)

It was also clear that some CRs found maintaining confidentiality difficult, particularly where information had significance for others and disclosure could be construed as in people’s best interest. In this way, CRs could be placed in difficult situations and ethical procedures had to be reiterated at regular intervals to ensure understanding and adherence by all.

CR involvement in the research had significant effects on their relationships with community members beyond the life of the project. CRs lived and, in some cases, worked in the communities under study and so encountered the research respondents repeatedly following completion of the study. In some cases this presented the CR with dilemmas. The process of conducting the research had allowed the CRs access to information about people’s illnesses and private lives that they would not normally be privy to. Following the research, it was difficult for CRs to know whether to pretend that they did not know anything about the issues that had been shared or to show concern and enquire after the situation. In a couple of cases, CRs reported that their friendship with particular individuals had been adversely affected. CRs also reported forming strong attachments to some respondents or feeling responsible for them.

I remember going home and saying to my wife that, you know, I’ve met this guy with a kidney problem and da, da, da, da … normally we wouldn’t discuss work, but I’m saying, “I think, you know what, I’m thinking of asking him to come and spend weekends, you know, just on the day, not sleeping over, but just come and sit by us and, you know, with the kids around” and, you know, and I’m thinking, you know, “Where am I going with this?” (CR2)

Some CRs reported feeling obliged to make telephone calls, pay visits or try to help in other ways following completion of the research, and some clearly found the situation distressing.

There was nothing that I could do to help them … They [community members] thought it’s good that I can put their points across and I thought it was good that they thought that, “Because she’s Pakistani, because she speaks the language, maybe our problems will get solved and she can do something.” (CR4)

In many cases, CRs felt that they had taken favours in order to complete the research, for instance by taking people’s time for interview or using their contacts or space to arrange data collection activities. The ongoing nature of their relationship with these community members implied the need for such favours to be repaid in some way. Thus, CRs ended the research study indebted to community members in a more serious way than the university research team, who could move on.

Being close to the community under study brought other complications too. One CR, for example, reported that community members had enquired about the state of her marriage and commented on her weight and that she found it difficult to counter gossip, suggesting that the role brought her into the community spotlight in a negative way.

The issue of monetary payment also brought dilemmas to the research process as payments to CRs could be interpreted by some as money for the ‘community’. In some cases, it was queried why the individual CR should benefit from something that related to the community more generally, and people also questioned how certain individuals had been recruited to the role. CRs themselves debated how fair it was to ask respondents to give their time for free (an approach that was adopted in order to be consistent with other activities ongoing in the locality by our partner community organisation), especially as many of those interviewed were from disadvantaged backgrounds. CRs told us that they would have liked to have been able to offer respondents something in return for their time.

I did feel a bit cheeky about that especially with the poorer communities because obviously most of these people aren’t working or … or part-time or low paid. It’s … to ask them to give their time for free … yeah, I did feel a bit cheeky doing all that especially when I knew that I was being paid for it, so … the most you could … be able to offer them was a cup of tea. (CR8)

### Role conflict and reflexivity

Perhaps unsurprisingly, we found that playing the role of CR was difficult for some since it conflicted with other local roles and identities that they already had. In particular, some CRs struggled to ‘step back’, to be reflexive and to acknowledge the importance of analysing their own preconceptions during the process of generating and interpreting data.

Several CRs were motivated to join the research team by their desire to address the disadvantage and suffering they observed in their communities. Several had worked on projects in the community and saw the research as a continuation of an ongoing personal investment. CR4, for example, commented that the role ‘did appeal to me because I was doing positive, helping the community, putting their voices across, taking down their experiences and how they deal with the situation’. While this commitment meant a highly motivated and hardworking team of CRs, problems arose when CRs saw their role as one of enlightening or changing community respondents. CR6, for example, spoke about a desire: ‘to educate the people [about a] normal lifestyle and improve their lifestyle.’

During analysis sessions, several of the CRs found it hard to acknowledge and question their own presumptions. Some were openly critical about community members and talked negatively about issues such as divorce, asylum, forced marriage, extra-marital affairs, community insularity, cultural change and poor standards of education, often appearing to blame individuals and families. For some, information gleaned through the research process was unwelcome and resulted in discomfort. Opinion-forming seemed difficult to resist owing, in part, to the closeness of CRs to the issues. Examples included one CR who felt unhappy about the revelation of divorce in the Pakistani community where she lived.

Women call their husbands over from Pakistan and how they spend 20 year … 30 years with them and how they [the men] go back to Pakistan and get married to another woman and divorce the first one. I think there should be some kind of law to stop that from happening. (CR4)

Importantly, also, we found that some CRs tended to essentialise their own communities and to exaggerate the differences between themselves and other ‘groups’. This was found to be a challenge during analysis sessions and was also reflected in some of the CR interviews.

Pakistani community is sometimes is … they don’t feel free to tell all their problems, but, you know, it’s to make them understand yeah … you know, it’s … Pakistani community, they always hiding the things like, you know, it’s … they don’t feel free to talk with anyone, yeah. (CR3)

On the other hand, some CRs had clearly thought about their new role and developed reflexivity during the course of the research. For instance, a female CR decided to dress in a very different way to usual in order to signal her new role to community members and, she hoped, elicit the type of engagement she expected. Others felt that they had learned a great deal about the issues facing members of their community, and this new knowledge had challenged some of their preconceptions.

CR1: I’ve learnt that my assumption that, you know, most Ghanaians are like this and that, but, you know, it has been challenged, you know, by the different

I: Did it surprise you?

CR1: It didn’t surprise me. I hit myself and I said, “But of course. What do you expect?” you know. We’re not in a kind of a stationary culture, you know. The culture is [transient], you know. I’m … you know, we … we’re going through many changes even in Ghana and over here. What they bring here and what they take out and, you know, there are all different kind of things and it challenged my mind a bit that … I need to broaden it out and, you know … and … so for me it’s … it has made me more aware.

### An empowering process?

The CRs reported many personal benefits from working on the project. For some, they felt that it had expanded their social networks and developed new skills.

I like to study, I like to learn so long as, as I said, it will improve my knowledge, skills, even my attitude. So I felt this was a good opportunity for me to, er, relate to them [other team members] and learn … and learn and get on well with them. (CR1)

There’s lots of things that I’ve learnt, lots of new skills I’ve learnt, lots of new tools I’ve learnt, lots of new communication ideas I’ve learnt; how to communicate effectively, what kind of language to use, what tone of voice to use and stuff like that. (CR4)

CRs also felt empowered by the feeling of being important to the research process. Many reported feeling ownership of the research, and themes of respect and contribution were highlighted repeatedly in the interviews when discussing the research process.

I feel that, you know, the research is dependent on us … if you like, we are crucial … we are very, very important to the research itself……..[I felt] 110 % ownership of it. (CR2)

It was different ideas yeah and they [university researchers] really respect it. (CR3)

Some also felt motivated to continue to contribute in their communities now that they had been sensitised to the issues.

It’s [even] spurring me on that, you know, as soon as I put to bed my dissertation, you know, maybe I’m going to look for something seriously, you know, to do around this area and setting up some kind of a voluntary organisation to [address it]. (CR2)

The extent to which the research could empower at the individual level was, however, limited by an inability to involve the CRs in all aspects of the project, both because of time and financial resources. In particular, outputs from the research came to fruition some months after the official end date of the project, by which time most CRs had moved.

 Furthermore, for some CRs, who had seen their involvement as a way of making a difference to their community, the lack of tangible impact of the research was a source of disappointment and frustration. In some cases, interactions with local people had heightened their awareness that the impact of the research would be less than they had hoped.

Some Bengali people […] ask, “What’s the benefit of this research project?” It was a new thing. A new question for me; “What’s the benefit?” But it is difficult for us to explain it. If we explain them in our way, according to our interview system, according to our aim and object, then they are not satisfied. They think no benefit … not benefit. Sometimes they say, “No benefit.” (CR7)

I don’t think without the research there would be any [change], so it is important to have the research. Whether the research will actually lead to changes I’m afraid I’m a bit cynical on that. Hopefully it will, but as I say, without it there definitely wouldn’t be. (CR8)

## Discussion

Our findings raise a range of important ethical and methodological issues, as well as practical considerations, for future public health research that seeks to engage CRs.

### Ethical considerations

The findings suggest that important ethical issues can arise when local people are charged with sensitive and time-consuming tasks such as the following: encouraging participation and gathering and interpreting data and representing their ‘community’ though research findings. Many of the CRs reported very positive experiences of taking part in the project with the researcher training, relationships with the university researchers and working as part of a team all cited as valuable. These findings mirror the accounts of community members engaged in other research studies [26, 27]. This is not to say, however, that positive accounts should be accepted uncritically.

It was clear that the CRs invested significant personal resource—material, emotional and reputational—in the project, owing to their ‘closeness’ to the individuals and communities recruited to the project. The involvement of CRs was extensive and raised the concern that an unreasonable responsibility was placed upon them to represent their community. Community members also tended to consider CRs to be responsible for the impact of the research at a local level. As Elam and Chinouya note, ‘People have to believe that the research will benefit their community. If the community … does not receive feedback or see any benefits, this belief will be difficult to sustain’ [28, p9]. As CRs were not in a position to effect change, these expectations presented a burden on both the CR and the research as a whole. Although some of the CRs evidently held rather pragmatic views in this regard, it was clear that social change was often a motivation for initial engagement. This suggests a need to address CR and wider community expectations to develop a more realistic understanding of the role of research evidence in tackling complex public health issues. As such, our findings support recently produced guidance for ethical practice in participatory research [29] that emphasise the importance of adopting sound principles and also developing appropriate training, support and monitoring systems to ensure best ethical practice in working with CRs. The present study, for example, used role plays and covered difficult situations in the research process as well as engaging the CRs in discussion on the social determinants of health and structural inequalities as part of the training process. Sessions on CRs’ own motivations for becoming involved in the research were also included, and CRs were encouraged to identify areas where they felt they might need support and guidance. Good ethical practice might also include the development of a peer support or mentoring network similar to those developed elsewhere [30]. It would also be advisable to follow the practice of Goodson and Phillimore [20] who included accredited training on community organising in their CR project as an added benefit of engagement and to promote personal development, a key motivator for the CRs in the current study.

One of the ambitions for inclusive work of this sort is to rebalance relations of power within the research process. As suggested by Maiter et al., the concept of reciprocity (implying co-dependency) in research practice can be a useful ethical principle in research of this sort [31] along with mutual respect and active learning [29]. In the current project, attention was paid to listening to and understanding CRs’ personal experiences and there was a commitment to their insights being represented throughout the research process. We adopted a combination of frequent contact between the university researchers and individual CRs at community level (for instance jointly undertaking visits to local places and interviewing local people together) plus facilitated group workshops in which all or sub-groups of CRs came together to share experiences and learning. This strategy appeared to engender a strong sense among the CRs of making a valued contribution to the project, as evidenced in the quotes presented above. We thereby achieved more than tokenistic involvement of community members [12, 32] and a partial shift in power away from university researchers to the CRs. However, drawing on Shippee et al.’s consolidated framework [17], it is important to note that, while the CRs were heavily involved in the execution of the study (design, recruitment, data collection and analysis), they had no engagement in the preparatory phase of agenda setting and limited engagement in the translational phase (dissemination and implementation). Difficulties arose at the point of completion of the project when contact with the university researchers effectively ended. At this point, CRs remain embedded in their communities and were still surrounded by the same problems the research sought to address. This suggests that rather than ‘exit strategies’, attention should be given to the development of ‘mobilisation for action’ plans [33] as well as more general ongoing support to CRs beyond project end. While we included dissemination workshops in which CRs reported on study findings, this activity was time-limited and one-way with no follow-on plan, highlighting the way that orthodox structures of research funding and governance limited the degree of empowerment and the phases and stages of the research that CRs could be engaged in [17]. A broader emancipatory and transformative impact at community level would require longer term collaboration between research professionals and community members and a shift towards a more participatory and pluralist mode of involvement [34]. This type of sustained, equitable partnership requires adjustment by funding bodies and necessitates applicants to more routinely see the value of, and make the case for, long-term engagement, core issues that are more fully explored by elsewhere [5, 35].

### Methodological implications—improving the ‘quality’ of research?

The generation of knowledge through inclusive research approaches raises fundamental methodological questions. Since the main project engaged CRs within an ethnographic framework, it is appropriate to draw on Hammersley’s ideas about how we should evaluate the quality of the research. It is useful to reflect on what the CR experiences suggest about the ‘validity’ and the ‘relevance’ of the research conducted with their involvement [36].

Considering first the question of ‘validity’, Hammersley suggests that any assessment of researchers’ claims should consider the characteristics of the research team and the ways in which the research has been conducted as well as the degree to which the evidence presented in support of these claims is convincing and plausible [36]. The findings above suggest ways in which CR involvement may both enhance and undermine the ‘validity’ of claims relating to the social determinants of health and health inequalities.

First, in relation to the volume and range of data generated, CR involvement was found to enhance our access to marginalised communities. CRs’ local knowledge increased awareness of who should be represented in the study, and CRs were able to access networks that might have otherwise been closed to research. Nevertheless, CRs reported finding it difficult to access all sections of their ‘community’—for example, class, sub-ethnic group and gender divisions—demonstrating the multiple axes along which identity situates. It is important to guard against CRs falling back on close friends and relatives to provide research responses and support CRs in recruitment strategies so that multiple points of contact are achieved and a diverse body of data generated.

Second, it cannot be assumed that CRs will be able to access more easily information about sensitive issues than professional researchers. CRs may face similar or even greater obstacles as outsiders if they are seen by respondents as being ‘too close’. Similar to Lundy and McGovern’s findings, some CRs suspected responses were sometimes guarded and incomplete [37]. ‘Insider’ status is also complicated by a broad range of social identifiers such as class, migration status, employment status, age, generation and gender. Understanding the effect of such multiple identifiers and interactions on the data generated is difficult and demands careful reflection among the research team.

Further, it cannot be assumed that CRs will necessarily offer a more nuanced and detailed understanding of the issue under question than professional researchers [21]. While the CR’s social position, personal biography and contextual familiarity did enable important insights, their ‘closeness’ could also result in normative interpretations and failure to consider alternative lines of explanation. Our observation that some CRs essentialised particular ‘groups’ and adopted normative discourses about ethnic difference reflect this concern and mirror other project experiences [20, 22]. Indeed, the ‘deep structures’ of society, including gender, class and racial hierarchies, may not always be visible to those who are disadvantaged, even as their oppressive effects are experienced on a daily basis. There can be a danger that the assumption of credibility can mean that CR analysis and testimony are left unchallenged rather than scrutinized alongside all emerging claims from the research process. Research adopting a CR approach will therefore usefully establish tools to encourage reflexivity and methods of challenging normative understandings and preconceptions in training as suggested by Ryan et al. [22]. Wright et al. usefully suggest that ‘intersubjective validity’ be considered a core dimension of quality in participatory research; that is the extent to which findings are credible and meaningful to different stakeholders from a variety of perspectives [29]. These issues also point to the need for openness and operational clarity from the outset. The adoption of deliberative strategies to acknowledge the skills, knowledge and contributions of all team members, and to establish principles of constructive challenge in the interpretation of evidence, will also be helpful.

A final point worthy of discussion in relation to ‘validity’, given the project’s focus on ethnically diverse neighbourhoods, is whether foregrounding ethnic identity was an appropriate strategy in the recruitment and deployment of CRs. Collective ethnic identity clearly held meaning, particularly for the minority ethnic CRs. Initial motivations and discussion of the importance of ethnicity in the research relationship highlighted the notion that ‘something would be lost’ if a shared ethnicity was not evident. However, the complexity of identity and its shifting nature militates against any assumption that ‘ethnic matching’ is possible and, as Gunaratnam would argue, is necessary or desirable [38]. We found that while shared ethnic identity was not meaningless in the research recruitment and deployment process, it did not necessarily imply increased credibility of claims and, as noted above, demanded careful reflexivity.

What then of the implications of CR involvement for the ‘relevance’ of our research? While accepting Hammersley’s recognition that research will often not have an immediate and direct impact on policy and practice, we concur with his belief that the rationale of (public health) research is to produce knowledge that has some public relevance [36]. The purpose of our larger study was therefore to generate knowledge that could inform more effective action on health inequality and poverty. It can be argued that the involvement of CRs in a research project, as with other types of patient and public involvement, may in and of itself increase the perceived authenticity, and therefore, relevance of the study findings to both professional and lay audiences. It was certainly our experience that the involvement of CRs was viewed favourably by our research funder and by our partner community-based organisation. Furthermore, conference presentations and academic papers based on the study findings have survived the peer review process, suggesting a perceived ‘relevance’ of the work in these circles. Nevertheless, it is hard to judge the extent to which CR involvement served to increase the study’s ‘relevance’ to different audiences or the likelihood of the findings being used to inform action. Further, as noted above, our failure to maintain the active engagement of CRs beyond the project’s end undermined the possibility of community members themselves being mobilisers of the knowledge generated into decision-making spaces, thereby, potentially increasing its impact on policy and practice. As such, we conclude that the degree of ‘catalytic validity’—another dimension identified by Wright et al. that refers to the extent to which new possibilities for social action are presented—was limited.

### Practical considerations

Handling the ethical and methodological issues raised above clearly relates fundamentally to the organisation and management of the research endeavour and the place of the CRs within this. We have already touched on a number of the structures and processes that we adopted, as well as some that appear to have been useful in other similar studies. Table 1 summarises these practical issues that are worthy of consideration by those embarking on public health research that engages CRs. These issues mirror and extend the themes found in a number of other useful publications on inclusive research and involvement [29, 39] and underscore the need to consider inclusive approaches ‘in the round’ when planning for research.

**Table 1:** Engaging Community Researchers in public health research: practical issues

Stage	Challenges	Useful practice
Pre-recruitment and recruitment	Representativeness: CRs may identify with a particular section of a community and will not be able to represent all community perspectives. Including people with the range of relevant characteristics such as gender, age and ethnicity can be hard.	Engage with community organisations and potential CRs at the time of designing the study
	Recruitment routes: using community organisations to recruit CRs may limit the pool of people who hear about the opportunity and the perspectives that are included in the team. Communities vary in their degree of collective identity meaning that CRs may be more difficult to recruit for some groups than others.	
		Establish a wide range of contact points and modes of advertising the role so as to reach all sections of the communities of focus
		Allow sufficient time for recruitment
		Involve appropriate local people in the selection process
		Include an interactive selection procedure that establishes applicants’ skills and experiences and gives them a chance to learn about the role
	Person specification: recruiting CRs with necessary skills and experience, especially cultural awareness and linguistic skills, can be difficult.	Understand people’s motivations for becoming a CR and how this might affect their engagement
		Ascertain the personal circumstances of CRs and how these might affect their engagement and need for support
	Payment: paying CRs can create conflict if other local people see this as unfair and think that the money should be ‘for the community’.	
		Plan to over recruit CRs so as to manage dropout and limited engagement
	Nature of contract: the irregular nature of work may be an issue for recruitment and retention, especially if it spans several months.	Agree with all stakeholders an appropriate rate of payment for CRs and ensure this is transparent.
		Carefully consider the pros and cons of compensating participants and agree an appropriate approach in consultation with all stakeholders
Training and capacity development	Developing and delivering a training programme: ensuring all CRs gain the appropriate skills, knowledge and confidence to undertake the role can be challenging when they come with a diverse range of experience, skills and education.	Use your detailed knowledge of the group of CRs to tailor the training appropriately
	Duration: finding a block of time may not be easy for CRs given personal circumstances and competing priorities.	
	Ongoing support and development: engaging CRs as part of the research team will mean a need for ongoing training and support throughout the project as different stages of the research unfold.	
		Include a comprehensive training programme that covers various aspects of the project from start to finish and is delivered in instalments throughout the project period
		Involve the CRs in identifying their learning needs, preferred learning styles and how they can support each other’s development
		Use a familiar and informal venue, flexible timing, limited paper-based work, interactive and informal modes of delivery, as these are likely to facilitate learning
		Ensure that any special needs are catered for such as linguistic, practical (e.g. child care) and cultural (e.g. space for prayers, halal food) factors
Retention	Competing priorities: Other job opportunities, health issues, personal priorities, and waning interest can impact upon CR retention.	Devote sufficient time for team building, establishing trust and mutual respect
		Be responsive to the needs of the CRs. Schedule in regular times and spaces for support and reflection
	Concerns relating to losing state benefits: CRs may drop out if they are worried about working too many hours or contravening other rules.	
		Be flexible with CRs’ working patterns and payment systems. Link CRs to sources of advice on benefit entitlements and regulations
	A stressful role: aspects of the CR role can be experienced as stressful, including: learning new information about the community, being in public in a new role, misunderstandings and conflict with community members, and completing a demanding schedule of work.	
		Have contingency plans so that individual CRs do not feel too much pressure to complete work alone
Conducting the research: supervision and ‘duty of care’	Coordination and completion of work: competing commitments can mean that CRs do not complete allocated work.	Formally agree contracts with CRs and set out expectations clearly on all sides
		Provide close supervision and support through informal meetings as this may help in identifying any problems early on
	Managing a diverse team: CRs may tend to associate with people in the team who they identify with (perhaps on the basis of a shared ethnic or other identity), and this can undermine cohesiveness and shared learning.	
		Provide ongoing attention to motivate CRs and demonstrate the value of their contribution to the project
	Boundaries in supervision: there can be a risk of getting over-involved in CRs’ lives and transgressing professional boundaries.	Ensure opportunities for CRs to voice concerns and problems; encourage openness, regular contact and approachability across the team
Collecting and analysing data	Conducting data collection: cultural norms, personal beliefs and contextual factors may make it difficult for some CRs to engage in particular data collection activities. For instance, they may find particular topics sensitive or shameful or they may face barriers to engaging with certain respondents.	Develop a detailed collective understanding of the socio-cultural, religious and political context within which the study is being conducted and how this may impact upon the CRs’ role
		Plan work allocation together with CRs rather than assume what is acceptable, convenient and doable
	Analysis and interpretation: CRs can struggle to be reflexive with normative understandings and preconceptions sometimes constraining the process of developing new insights.	
		Allocate work so that it takes account of the strengths, weaknesses and personal attributes of individual CRs
	Outputs and dissemination: project timescales and the need for efficiency can often make it difficult to involve CRs in report writing and production of other research outputs.	
		Include time and opportunities for developing reflexivity. Assess the findings explicitly in terms of the extent to which they are credible to different stakeholder perspectives
		Cost research projects realistically to ensure that CRs can be kept involved right through to the dissemination and implementation phases
Beyond project end	Lingering negative effects: CRs may feel disappointed as the project comes to an end. CRs may also feel indebted to community members and may face negative reactions, particularly if there is little tangible impact of the research at community level.	Be aware of potential unintended negative impacts of the research and ensure that the training provided to CRs equips them to avoid or manage such situations
		Include debriefing sessions that reinforce the need to maintain confidentiality beyond the end of the project
		Explore the potential to develop ‘mobilisation to action’ plans that can be operationalised by CRs beyond the end of the research period
		Contribute to the continued professional development of CRs by connecting them to other opportunities and accrediting their involvement in the project

## Conclusions

The insights presented above confirm that paying close attention to the accounts of CRs is instructive in understanding the strengths and weaknesses of such inclusive research endeavours. The study revealed a number of practical lessons, most of which centre on the need to establish ways of working within the research team that provide space and time for reflection, mutual exchange of ideas, development of trust and collective problem-solving. The findings highlight various ways in which CR involvement can enhance the ‘validity’ and ‘relevance’ of research but also caution against assuming that such increased quality will be necessarily forthcoming. CR involvement raised novel challenges for the professional researchers and underscored the importance of adhering to standard principles of reflexive and ethical research practice. The CRs generally reported very positive personal experiences of being engaged in the research team, despite being frustrated by the limited ability to enact change through the research. The sense of ownership gleaned from participating in the research enhanced personal skills and, to some degree, community capacity. We conclude that for research of this type to meet its full potential and avoid harm, there is a need for careful support to CRs and long-term engagement between funders, research institutions and communities. Research participation should not be viewed as the ‘silver bullet’ to exclusion [40] but a valuable asset in the toolbox of methods that can contribute to positive social change with the aim of reducing health inequalities.

## Footnotes

1 The word ‘community’ is used rather loosely here, and we recognise that it is not an unproblematic concept. Depending on the research context, ‘community’ can refer to a geographically delineated area or settlement or to a group of individuals who recognise shared membership that could be based on a variety of social identifiers such as ethnicity, occupation or disability.
